# “I’d Probably Be Homeless”: Basic Income Participants’ Lived Experiences Related to Housing Stability, Health, and Wellbeing

**DOI:** 10.3390/ijerph23040417

**Published:** 2026-03-26

**Authors:** Ahna Ballonoff Suleiman, Selena Regalado, Emmanuel Onuche Momoh, Katherine Menendez, Catherine K. Brinkley

**Affiliations:** 1 Center for Regional Change, University of California, Davis, CA 95616, USA; stregalado@ucdavis.edu (S.R.);; 2 Department of Human Ecology, University of California, Davis, CA 95616, USA; eomomoh@ucdavis.edu (E.O.M.); ckbrinkley@ucdavis.edu (C.K.B.)

**Keywords:** basic income, guaranteed income, qualitative methods, housing stability, wellbeing

## Abstract

**Highlights:**

**Public health relevance—How does this work relate to a public health issue?**

- Poor physical and mental health outcomes are associated with living in poverty and reduced income generation.
- Economic hardship can contribute to psychological distress and negatively impact parenting practices, and ultimately, the health and wellbeing of children.

**Public health significance—Why is this work of significance to public health?**

- Basic income programs for families with young children can offer a reprieve from poverty that may have long-term benefits for both parents and children.
- Basic income funding can enable housing stability, which is essential for child health and wellbeing.

**Public health implications—What are the key implications or messages for practitioners, policy makers and/or researchers in public health?**

- Time-limited basic income programs offer families offer critical reprieve from poverty and they do not, on average, provide sufficient resources for families to achieve long-term financial stability.
- Basic income programs give parents time and space to think, plan, and attend to the health needs of their children.

**Abstract:**

This research draws from participant interviews at baseline, midpoint, and conclusion of a two-year Basic Income program designed to lift 76 families, with at least one child under the age of six, above the California poverty line by supplying a guaranteed monthly cash stipend (average: $1289 per month). Theoretically, we bring the Family Stress Model into the conversation with the Theory of Change that underpins Guaranteed Income programming, namely that freedom and choice empower families to mitigate stress, manage funding, and better navigate the multifactorial aspects of living in poverty. Participants report that the Basic Income program offered a much-appreciated reprieve from poverty and reported using the funds to stabilize their housing and support the health and development of themselves and their children. Participants also highlighted how guaranteed cash programming can pair with traditional social service case management to better benefit recipients.

## 1. Introduction

In the United States (US), poverty affects 39.5 million (10.6%) of residents [1]. In California, 13.2 million (34.8%) residents live in (16.9%) or near (17.9%) poverty [2] and struggle to find housing amid an ongoing housing crisis [3]. The US childhood poverty rate rose from 5.2% in 2021 to 14.3% in 2024 [1] and California’s child poverty rate rose from a historic low of 7.5% in 2021 to 18.6% in 2024 [2]. While researchers have documented multiple associations between poverty and behavioral, structural, and political factors, studies demonstrating reliable and consistent causal relationships have been elusive [4]. As a result, no one single solution, including unrestricted cash, offers an easy end to poverty.

Poor physical and mental health outcomes are associated with living in poverty and reduced income generation [5]. Americans living at the lowest income levels are over four times more likely to report fair or poor health status compared to people living at the highest incomes level [6], and recent studies show that adults with the lowest income and educational attainment levels have increased rates of congestive heart failure, heart attack, stroke, anxiety, and depression compared to wealthier, more highly educated peers [7,8]. Children who grow up in poverty exhibit differences in structural and functional brain development compared to children in higher-income households, which have been associated with disparities in academic performance, language acquisition, executive functioning, and social–emotional skills [9]. Correlational evidence reveals associations between household income and benefits for children, including increased educational attainment, improved school attendance, and greater use of preventive medical care [9].

In part, the Family Stress Model (FSM) explains how economic hardship creates pressure on caregivers, leading to psychological distress, which negatively impacts parenting practices and, ultimately, the wellbeing of children [10]. The FSM highlights how daily financial strains and stressors impact family processes, affecting mental health and child outcomes [10]. According to the FSM, as poverty exhausts parents’ emotional and relational resources to counteract crises as they arise, their parenting ability is compromised [11]. Following this model, many poverty alleviation interventions focus on alleviating poverty in early childhood to improve parental functioning and childhood health and wellbeing, but more research is necessary to understand whether the financial and housing stability provided during this time results in sustained benefits throughout the life course [12].

Seeking to fill the gap in federal and state programming and influence the critical development window of early childhood, several guaranteed income (GI) programs have provided families with unconditional cash payments for a specified period. Unlike many federal and state benefit programs, GI recipients do not have to repeatedly demonstrate eligibility or meet requalification requirements and can rely on steady funding over a specific time frame to address their personalized needs [13,14]. As a subset of GI, Basic Income (BI) programs offer enough guaranteed cash to cover a baseline standard of living for a given time. While GI participants may or may not experience poverty while receiving a set monthly stipend, BI seeks to explicitly boost recipients above the poverty line and meet basic needs with guaranteed cash.

Research on the impact of GI programs has measured a myriad of diverse outcomes [15]. A growing body of evidence highlights the potential for GI to serve as a powerful tool in addressing housing instability (e.g., [14,16,17,18]). Similarly, research suggests GI programs have the potential to mitigate the effects of poverty on the health of children and adults (e.g., [19,20]). Participants in GI programs also report improvements in mental health, overall wellbeing, and feelings of joy as families experience a reprieve from a life of poverty and experience gratitude for the temporary relief from poverty [21,22].

A foundational hypothesis of many BI programs is that providing families a reprieve from poverty during specific windows of child development may have long-term benefits for both parents and children [23]. While robust evidence demonstrates that BI participants experience a reprieve from poverty while receiving the BI stipend, most evaluations with long-term measures indicated early benefits attenuate or disappear following the cessation of payments [24,25,26]. This suggests that a temporary reprieve from poverty may not be sufficient to sustain long-term effects or improve child health and development [27].

This qualitative case study explores the experiences of some of the most vulnerable families with children under the age of six while they are receiving the BI subsidy. The two primary research questions for this project were: (1) how does receiving BI impact housing stability?; and (2) how does BI impact child and parent health and wellbeing?

## 2. Methods

The Yolo County Basic Income (YoBI) Program provided low-income families enrolled in California Work Opportunity and Responsibility to Kids (CalWORKS)—a cash aid and supportive services program for low-income families—basic income during the first six years of a child’s life. During March 2022, a total of 81 families were invited to participate in the YoBI. All adults that had at least one child under the age of six and were enrolled in CalWORKS and in the Housing Support Program (HSP) (N = 51) were invited. Based on available funding, an additional 30 adults with children under the age of six receiving CalWORKS but not HSP (out of a total of 343 eligible families) were invited. Five families did not respond to the invitation or declined to participate.

From April 2022 through March 2024, a timeline of 24 months, the remaining 76 adults received monthly, unconditional cash support in addition to their existing CalWORKS and other benefits, to bring their family up to $1 above the California Poverty Measure Minimum. The average monthly family stipend was $1289 per month. Household size ranged from two (one adult and one child) to eight (average = 3). Every household included one child under the age of six and the total number of children under the age of 18 in each household ranged from one to six (average = 2). Most recipients were female (92%) and single parents (68%). All but one of the single parents were female (98%) and one quarter (25%) were first-time mothers who were navigating the financial responsibilities of parenting and adulthood for the first time. Additional self-reported demographic information about participants is provided in Table 1.

There were two groups of participants: (1) YoBI + HSP received BI and housing wrap-around case management; (2) YoBI only received BI only. Participants who received CalWORKS and HSP experienced more significant housing instability than those who received CalWORKS only. We did not observe distinct themes between the narratives from participants in the YoBI only and the YoBI + HSP groups, with the exception that the YoBI + HSP participants provided insights on the additive benefit of housing support services, so we analyzed the data as a single group and coded themes from case management + BI as a standalone portion of analysis.

All participants received detailed information about the IRB-approved intervention and research and provided written consent to participate. Participants were invited to complete interviews at three timepoints: baseline (April–June 2022) when approximately two-thirds of the participants had not yet received their first stipend, and the remaining participants had received at least one monthly stipend; midpoint (May–August 2023) after participants had received BI for 12–15 months; and conclusion (May–July 2024) after participants had received their final stipend in April. At each time point, we made three attempts to contact people for an interview. Most participants (n = 66; 86.84%) completed at least one interview. Nineteen (28.79%) interviewees participated at all three time points and the remainder participated once (n = 22; 33.33%) or twice (n = 25; 37.88%). Occasionally, we reached participants and they declined or scheduled and did not show, but for the most part, the people who did not participate did not respond to our invitations or provide an explanation for their lack of participation. Participants received a $50 Walmart gift card after completing each interview.

Members of the research team with expertise in qualitative methods for unhoused populations developed the interview guide, including questions about participants’ lived experiences receiving the stipend. At baseline and midpoint, peer interviewers, who had lived experience with poverty and had recently received welfare benefits, received training from research staff and conducted the interviews. Based on feedback from the program team and peer interviewers, we shifted strategies and members of the research team with training in qualitative methods, most of whom had relevant lived experience, conducted the third round of interviews. Each interview was conducted in-person, in English or Spanish, lasted between twenty and forty minutes, and was digitally recorded and automatically transcribed using Microsoft Teams or Zoom. The research team used the audio files to verify the system-generated transcripts, translated (when necessary), and used Dedoose to code the transcripts.

We used a modified inductive analysis approach to analyze the data [28]. This involved a cyclical process of identifying and exploring emergent themes, coding the interviews, condensing the raw data into key themes, establishing links between the original evaluation objectives and the summary findings, and synthesizing the experiences and processes that emerged in the narratives [28]. After each round of interviews, the team collectively developed the code book by reading a subset of the transcripts and discussing the emerging themes. After coding all interviews, we revisited several key themes that arose from the data that aligned with the research questions and the existing evidence on BI projects. As themes emerged, we extracted excerpts from the transcripts and verified the original codes. Due to the consistency of the themes across the different time points of interviews, we present an integrated analysis of all themes except for the concerns about the end of the program, which appeared only during the last set of interviews. The baseline data was collected by the Yolo County Health Department independently. The University’s Committee for Protection of Human Subjects reviewed and approved the study protocol and interview guide (Protocol 1789301-1, approved on 22 February 2022; Protocol 1789301-2, approved on 16 August 2022).

## 3. Results

Here, we present the narrative themes that emerged from the baseline, midpoint, and endpoint interviews.

### 3.1. Baseline: The Promise of YOBI

At baseline, participants described the significant hardship of living in poverty and the hopes they had for BI to offer them some relief. Approximately two-thirds (66%) of the participants were unemployed. The main reason that people struggled to find and/or maintain consistent employment was the challenges of finding work that aligned with or accommodated their children’s school and/or childcare schedules. People also attributed employment instability to the COVID-19 pandemic.

Participants described being overwhelmed with paying utility bills, car insurance, gas, food, diapers, clothes, and other essentials for their children and households. As one mom articulated, “I feel like it’s very stressful. Like I’ve never lived on my own and I’m … learning how to be an adult … I feel very overwhelmed. It’s a lot”. Overall, participants desired secure, adequate housing and faced challenges achieving that goal amidst financial constraints and personal hardships. People planned to use BI to cover costs associated with housing and transportation. Many also spoke about the importance of saving money, building credit, and even purchasing a home to create a stable future. As one participant envisioned: “I’m trying to … build a legacy and you know, and my self sufficiency … build my credit and pay off my credit cards and … save for my house.”

Participants’ current housing situation heavily influenced their future plans. While some people rented and hoped to own, others who were unhoused or living in temporary housing aimed for permanent housing or safer living situations. One participant described moving into affordable housing as “… a transition from homelessness, but it’s insufficient living, it’s not … enough rooms for all of us and it’s a little rough around here”. Lack of secure housing was a primary source of stress impacting all aspects of parents’ lives, including their capacity to feel confident in their ability to care for their children. As one participant describes:

“… I have nowhere to go that’s mine and everybody cramped in that room. And stuff’s falling off that’s all over the place because it’s backed up in a hotel … It affects my mothering … it affects my relationship with myself … it affects everything right now.”

A primary motivation for having stable housing was to ensure their children’s wellbeing. People reported that being unhoused or having unstable housing created stress for them and their children. The potential of receiving BI offered the hope of some reprieve from housing instability. As one person envisioned: “I think I will be able to do a lot more for me and my kids, not only … just survive, but be able to live life and be comfortable.” The participants who had received one or more BI payments at the time of the baseline interviews described how it had already helped them transition from homelessness and the immediate relief that they felt. As one parent describes, “I’m in a motel instead of in my car with my daughter … it’s [YoBI] got me where I needed to be”.

In addition to directly covering housing costs, participants also expected that the extra income would help them pay rental application fees and increase their eligibility for better housing. As one participant shared, “My housing worker assistant says this is gonna help me because it’s gonna be in my income that I can show … [to help] me get into some more like low-income housing”. They also predicted that BI would help them reduce their stress. Single mothers particularly talked about the potential of having this extra income, with one person describing it like having an extra income-earning parent or adult in the house. Many people described a tension between wanting to save the BI funds while also paying off debt or covering immediate expenses. Some participants had long-term goals for saving the funds but were not clear about how they were going to realize those goals.

### 3.2. Use of BI Funds

Throughout the program, participants used the funds to cover their basic needs. Participants used the funds to cover housing, transportation, clothing, health care, childcare, child enrichment and development, and daily living expenses. Many families had never had discretionary funds and described using extra income for fun experiences, clothes, or opportunities for their children. Two participants reflected: “The priority was always rent and essentials, like food and transportation. After that, if anything was left, we used it for fun.” and “… clothes for my kids … some entertainment activities for them like the movies … give my son a little extra allowance … be able to buy clothes, able to get out of the house because we had gas.” Almost everyone discussed using the funds to secure reliable transportation, including purchasing more reliable cars, repairing existing cars, paying car insurance, and buying fuel to get to childcare, school, work and other essential destinations. Participants often remarked that having unrestricted funds to cover these expenses was a unique benefit of BI.

Some people reported being able to save enough money for emergency expenses. Many appreciated having a bit of a safety net for unexpected expenses—as one participant describes: “It’s helping me save just in case anything happens with this job or one day services get cut out of nowhere.” Most participants used the money to pay off debts and some were able to save small amounts that allowed them to budget and prepare for the future expenses a one participants highlights: “I’ve never, in the past, never been able to save money, and I’ve always been like in debt, and I had claimed bankruptcy in 2020. So I was pretty much at zero … So it helps me save” and another echoes:

“Definitely a lot of saving, a lot of putting things to the side so that I can have something for backup later in case of emergency … a lot of just trying to get things in on time and not end up having to owe anybody.”

A few outliers were able to save money to help pay for school, start a college savings account for a child, or put towards a down payment for a new home. A small group of participants reported using the money to cover educational expenses as they worked towards academic degrees that would help them better support their families. As one participant who was a full-time student described: “Buying my books … There are three to four hundred dollars … I had to buy new laptop for school … everything adds up … if you’re not working. Daycare on the weekends, if I had to study more and I have to do that …”.

### 3.3. Benefits of YoBI

As hypothesized, participants experienced benefits while they received the BI payments. Many participants had always lived in poverty and BI allowed them to experience life with greater financial stability. As a result, they were able to see a different future for themselves and their children. Parents also said that having BI reduced their stress levels, which made them better parents to their children. Collectively, participants described how the reduced parent stress coupled with the material support positively changed the childhoods of the children in the program.

Unanimously, participants reported that having cash assistance was helpful and expressed significant gratitude for receiving BI. As two participants summarize: “YoBI’s been a huge blessing” and “It helps me stay afloat … it keeps me from drowning.” Participants highlighted the ways in which it reduced their feelings of stress and increased their feelings of gratitude. Some participants described how the cash assistance was particularly helpful for families who had the most restricted financial resources.

#### 3.3.1. Housing

Participants reported stabilization in their housing and used the money to cover rent. They also described using the money to demonstrate income on rental applications. As one participant reflects:

“… the only reason I was able to get into my apartment that I’m in now is because I was able to claim that income. Like without it, I wouldn’t have qualified. So that kind of saved my life because there’s no way I could afford the rent where I was at any longer.”

Participants also use BI to cover other housing-related expenses like insurance and utility bills. Many people directly attributed their housing stability to BI. As one participant summarizes: “YoBI … has been a great deal of help. Without that last month, I wouldn’t even have been able to pay rent.” and a second explains “I’d probably be homeless. I’d probably be out there looking for places to sleep on … so definitely would be super low on money if I didn’t have it [YoBI]”.

#### 3.3.2. Child Health and Wellbeing

A focus of this research was to explore the ways that BI impacted child health and wellbeing. Overall, participants reported that BI positively changed their children’s lives. At times, participants described a direct impact of BI on their children’s wellbeing, such as being able to access health and educational services. Some participants described how having more financial stability also allowed them to show up and engage more proactively in their children’s schooling. As one participant articulates, “This program has really helped me focus more on his [my son’s] mental health and his learning”. Another participant described the value of having time to authentically partner with her child’s educators:

“I was able to figure out exactly what was going on, and be able to show up to his school, and, you know, just be more involved with the teachers and counselors and stuff that he has to deal with on a daily basis.”

The parents in the program who had children with special needs, including ADHD, learning differences, autism, genetic disorders, or other medical needs, found BI particularly helpful. Some parents described that BIs’ impact on their housing location or stability helped their children. As one participant observed: “My middle son has … autism, along with his genetic disorder. And since we’ve moved … he’s actually thrived a lot. He is doing very well in school”. Other people described that BI allowed them to have the flexibility to attend to their children, and access care and services. In describing the challenges she faced retaining stable employment with a child with additional needs, she shared: “When he [my son] is having a bad day … the daycare won’t take him, you know. So that’s more time that I spend with him at home … I can’t work.”

Participants also described how the reduced financial stress allowed them to spend more time engaged in activities with their children. They described increases in both the quantity and quality of shared time. As one participant shares: “… the kids … got stuff that they wouldn’t have gotten before YoBI and there were … better birthdays … they got to … go play soccer … this weekend we … went bowling … it changed their childhood.” A second participant explains: “I had more help to pay the rent, so that allowed us to have more family activities.” One of the greatest benefits participants reported was that the additional financial support afforded them more time with their children. As described by one participant: “We actually spent more time … together”.

The majority of the participants discussed trying to figure out how to balance work and parenting infants and young children. At times, employment felt like it didn’t make financial sense. The costs that participants had to pay for childcare sometimes exceeded the amount they were able to earn, particularly if they had multiple children at home as summarized by one participant, “You work to earn money, but you also have an additional expense, childcare”. Participants said that it was often difficult to find a job they could do that matched the time constraints of childcare or school schedules. One participant pointed to the challenge of finding work that matched her child’s schedule:

“I don’t really have a lot of time while [my son] is at school … I don’t work because it’s kind of hard putting an application and then they’d be like, well you only got four hours and we need more throughout the day …”

As a result of these challenges, participants often expressed the value of BI as being able to stay home and care for their children. Parents expressed concerns about finding childcare providers they felt they could trust and described how this affected their choices around employment. Finding work that was flexible enough to accommodate child care schedules was a persistent challenge. As one participant indicates: “My main reason for not working right now is … I do not have trust in people to watch my kids so … I’d much rather stay with them as long as possible.” Even when they were able to find reliable childcare, participants worried about the wellbeing of their children. As one participant shared: “I have bad anxiety … I quit two jobs, like because I couldn’t … do it, I had to stay home with my baby.” Every participant talked about the way in which BI alleviated their stress. One single mother highlighted this benefit:

“I was able to stay home. I’m a single mom so it helped, so that I can not work as much, so I can be with him. Just have more time with him because he’s so young, and I feel like he needs that.”

Other participants reported an indirect impact as they attributed improvement in child wellbeing to increased housing stability they achieved as a result of BI. Sometimes they attributed this to general improvement in wellbeing and behavior. As one participant reports, “… my daughter, she … is thriving since we’ve been in this home … It really gives her a sense of stability and comfort, and just everything has been great.”

#### 3.3.3. Parent Health and Wellbeing: “It Helped Me Feel More Secure—Okay, I Can Do This.”

Parents described many ways that the program improved their health and wellbeing. Overall, people felt significant benefit from the self-reliance they experienced in the program. This was particularly true for younger, single mothers. Many participants described being able to live independently for the first time as illustrated by quotes from three different participants:

1. “I didn’t love myself like until now, the money that you gave me.”
2. “I had to learn to be independent … it helped me grow as a person”.
3. “YoBI kind of gave me that boost and that confidence I needed to provide for my family and I was able to move up in my life. It was my position confidently, knowing that I was secure, you know.”

For many participants, this was the first time they felt confident as parents: “For the first time in my life I was able to feel confident in my ability to be a mom and provide and I wasn’t worried and stressed about, ‘how am I gonna do this?’” This shift in their self-perception and their ability to cope was a universal theme in the interviews. One participant explained:

“I was able to … be a better mom for my kids, cause I’m always stressed with bills and trying to figure things out. But getting that money, it gave me a better mental state for my babies, so I can focus on being a mom, being more kind and patient.”

The extra financial resource provided important emotional support. Some single parents described BI as another income in the household. One participant states: “It’s … pretty much my other co-parent.”.

#### 3.3.4. “Our Future Has Changed”

All but one participant reported that receiving the funds changed their lives for the better. The increased financial security offered participants to envision a different life for themselves. Reflecting the feelings many described about the program offering some reprieve from the struggle, one participant shared:

“… I was in the dark, basically struggling all the time. So when I came to this program, I really opened my eyes … life can be better … more, you know, positive instead of just in stuck in this dark hole. … It got me out of that survival mode. And now I’m just living life instead of struggling.”

In addition to the immediate benefits, they also described how the relief from extreme financial stress changed the possible future they could envision for themselves and for their children. As one participant captured: “YoBI gave me an insight of what life could be … When you … [are] growing up in the situation that I did, where you … go without so much, you feel like … it’s normal to struggle.”

Participants also described how BI changed their attitudes towards work and their motivation to earn money. Having more financial resources gave people a taste of how their life could be different and left them wanting more. As one participant described:

“I was like, down here with my money. Now I’m up here …. I don’t want to go down again … How would you say that you are wanting to be more ambitious in the future because I have lived through what I have lived? … It gave me satisfaction to be able to save money, even just a little …”

While some participants described how the program allowed them to be home more with their children, others reported that being in the program and having more financial resources motivated them and provided them with material resources, such as reliable transportation, to seek more employment opportunities. As one participant explained:

“… when I first got the YoBI supplement, I was told this is to help you get better in life, to get a job and get your monthly income … so you can get further. So, that really motivated me to get a job. So, I got a job and it really does help and it does make me feel a lot better about myself and towards my kids.”

For some parents, this motivated their actions to return to school, get additional training, or find a more stable job. Thinking about her experience, one participant noted: “It [BI] just makes me want to go to work. It made me realize that it’s better to live above the poverty line … which is why I went back to school”.

In sum, participants identified many benefits of this program. They often described the program as a blessing and expressed that they wished that others could also have the opportunity to receive BI. They often said that BI arrived at a critical time in both their and their children’s lives. They described ways in which they felt that it directly and indirectly improved the health and wellbeing of their families and provided them with essential, unrestricted funds that allowed them to best meet their unique basic needs.

### 3.4. Challenges of YoBI

While participants reported many benefits and expressed gratitude for the unrestricted financial resources, they still experienced challenges. For most of the families, the money was helpful during the two years of the project and it was not sufficient to permanently transform their financial situation. During the first year of the project, almost all participants expressed that they still experienced significant financial stress. Many entered the program with debt or other financial burdens. One participant shared a common sentiment: “My [first] year with YoBI has been a lot because … I had a lot of bills I was behind on, so this last year has been just trying to catch up with them and then pay everything off.” Participants reported spending money on rent and other basic needs, but often those needs exceeded the available income, even with BI. When everything felt essential, making the decision on what to spend money on was often challenging, as one participant describes: “I don’t think my spending was bad, because everything I spent it on was like, that’s the best thing”. Another participant explains: “It still wasn’t a lot of money you know, so we prioritize. Our rent was first … and then … prioritized by what was necessity [sic]”. For the significant majority, participation in this program was not sufficient to create a path to financial independence.

While BI reduced some of the immediate urgency around money and allowed families some stability, most participants still had unmet financial needs. They often described making progress in paying off debt and covering predictable expenses but faced challenges when faced with something unexpected like a car repair bill, medical need, or loss of employment.

“Even like with the two incomes … this whole check was just rent … that only goes so far … and then things come up … It’s like you have to prioritize what you have and there’s always going to be some that pops up … your 6 month insurance is due … car would break down … that really took all of our money.”

Many participants described that they made efforts to save funds throughout the program, but often found that a major life change or expense would quickly deplete their savings. As one participant explained:

“I saved it about a year and a half or so, and then I just couldn’t save it no more, I had to use it for my rent because … my husband wasn’t working. So all that savings was gone in like 4 months.”

The immediate need to maintain housing security was the primary reason participants gave for using up their saved funds. At the end of the program, many people wished that they had saved more money while they were receiving BI but also acknowledged that their day-to-day expenses made that challenging. As one participant noted:

“I started to put money aside … so when it was over, I probably ended up with an extra $600 … So I was able to have like an extra month of funds because I was able to save it throughout that time. If I could go back I would have been doing that the entire time. But again, the way life worked it just wasn’t possible, you know.”

Many participants expressed that while they planned to save money, the immediate emotional benefit felt from paying their bills, renting a more expensive place to live, taking their child out for dinner, or buying their child a new pair of shoes outweighed the long-term emotional benefit of saving. As one participant reflects:

“I should have saved some of my money that I was receiving, but I had just moved into a place … and then just gotten custody of my son … it helped me in a lot of beneficial ways, especially when I was homeless living in the hotel room. It definitely helped get things that I hadn’t had like clothes that fit right, and shoes …”

While participants clearly described increasing housing stability while they received BI, many also continued to encounter persistent challenges finding secure housing and paying rent on time. At the project midpoint, none of the people who were receiving YoBI + Housing Support reported being unhoused. Some of the participants who were not receiving housing support reported being unhoused but in improved situations, as one participant shared, “I’m homeless but I was homeless before the program also and it’s coming together”. Participants in both groups talked about the fact that more urgent, essential expenses consumed most of their financial resources, which meant that they still struggled to pay rent when it was due. One participant explained: “… even people that have money don’t have housing, you know, because they’re too busy still paying this other stuff in their daily life that they can’t afford or find housing”. Many of the participants who talked about BI providing housing stability reflected that they did not have a plan for sustaining housing stability after the last payment and were concerned about how that would affect the stability of their family. One participant reflected:

“[Without BI] I would probably be homeless … because I couldn’t afford to move out of where I was living, let alone afford the rent that I was paying to live in my old place. So I probably wouldn’t have my kids because I’d be homeless. And I’d lose my car. I would have … I would be literally on the streets. It would be scary.”

### 3.5. Need for Additional Types of Support

Given that this was the first time that many participants were living with enough financial resources to cover their basic needs, many participants expressed wanting additional support and services alongside the cash benefit. In talking about the program, one person reflected: “it helped me pay rent, but it was very superficial”. While financial counseling was offered to participants, few took advantage of it and many offered suggestions to improve the program:

“$1400 is a lot for somebody who has nothing. And when you tell somebody who has had nothing for a really long time, and you give them that amount of money with no skills … What do you expect to happen? Even just breaking it up into two monthly payments. One at the beginning of the month and one at the end of the month to-to help the struggle would have helped instead of, bam second Friday of the month, let me just drop you a heavy check. And leave you to figure it out … I wish that there would have been training or some kind of something about budgeting at the end of the day.”

Participants universally appreciated the advantage of having unrestricted financial resources that allowed them to pay for the things most urgent and relevant to their personal situation. They were not asking for advice on how to spend their money but they did acknowledge that they did not have the knowledge, skills, or experience to manage or save their funds. For some people, the need for financial planning support became particularly salient at the end of the program, as one participant said, “I needed help budgeting because YoBI was ending, and I had become dependent on it”.

### 3.6. End-Point: Effects of Losing BI “I Have No Nest Egg. I Have No Emergency Funds, Nothing to Fall Back on.”

At both baseline and midpoint, participants talked about the temporary duration of BI. Throughout the program, participants described efforts to make sustainable financial decisions related to housing and transportation that they felt they could maintain after the end of the BI program. One participant clearly articulated this: “I wouldn’t want to like put too much on … that income because it’s not forever … I feel like if I were to move into a bigger place financially when it’s gone, like how could I afford that?” This uncertainty about sustainability created stress for some participants. As one participant shared:

“Being independent causes a lot of stress, especially knowing that eventually I’m gonna have to get off of all this help. So that’s like a big worry all the time in the back of my mind because I’m thinking you know, I don’t want to go back to being homeless because I was homeless while I was pregnant. So I don’t want to … do that again with the baby.”

The concerns participants had during the program were quickly realized after they received their last payment. During the endpoint interviews, only weeks out from their last payment, participants were describing significant financial uncertainty and stress. Many participants were facing housing insecurity and were unsure how they would pay their bills. In the endpoint interviews, almost all participants shared uncertainty about how they would pay rent for the next month. One participant articulated this sentiment: “My rent’s not paid, and I have $13 to my name, and I don’t know how I’m gonna come up with it.” and another reinforced it: “I’m always on the edge. My rent’s not even paid for this month … my landlord checks me on Sunday for an update on the rent, like, I don’t have an update for you, cause I don’t have nothing.” Those who had managed to save during the two years of the program were quickly watching their savings disappear without having a clear path forward for sustained financial stability.

Participants shared emotional stories about the impact of losing the BI funds as they struggled to pay their bills. One participant described this saying “you know … we’re back to square one again without YoBI. We’re back to bills up high again”. While participants valued the reprieve from extreme financial hardship, some people also described that having the knowledge about what it was like to have more financial resources increased the disappointment and negative feelings. One participant shared: “It’s tough … knowing what it’s like to have more money than what I have”.

This uncertainty and instability created fear and anxiety for many people. One participant expressed: “I can’t … buy clothes for my children … and the food too. It’s difficult for me. It scares me a little bit … a fear that we are not going to be okay.” Another participant shared: “I just feel like I lost a husband even with YOBI. I feel like I lost that like I had some kind of you know something to lean back on”. Many participants cried during their final interviews as they described how they were feeling. One participant described the shift in her emotions, stating:

“Normally, I’m a strong individual. I don’t break easy. I don’t cry all the time. But here lately the last few weeks has been like, everything makes me cry like I’ve been so stressed, so like, overwhelmed, like, mentally, I just wanted to give up.”

Almost every participant described how they had to cut back as highlighted: “So we have to rethink everything and how to do things, save up more money, cut back”. Many participants described trying to figure out how to earn enough money to fill the gap left by losing BI. Parents struggled to earn enough money and cover their children’s material, emotional, and childcare needs. For a number of people, the end of BI coincided with another life transition such as loss of a job, beginning a new school or training program, sickness of a family member, or changing in housing stability that amplified the impact of losing BI. As one participant explained:

“it’s like it’s a challenge now … I’m on a work entry, so I’m not getting my normal pay … My income is less than my monthly bills. Now you know what I’m saying … This is the time I need it [BI] the most.”

While participants were all grateful for the reprieve they felt while receiving BI, they also described disappointment because they had experienced life with greater financial stability and now were returning to their lives of financial insecurity.

## 4. Discussion

Participants in the YoBI program largely achieved short-term improvements in housing stability, health, and wellbeing. Recurrent themes of hope in early interviews showcase how participants took actions that they would not have otherwise taken without BI. As previous studies have noted, the causes of and pathways out of poverty are complex and multifaceted, requiring a combination of strategies that are often tailored to individual experiences. Participants reported that BI gave them room to think, plan, and envision—which is necessary as people navigate the complexities of poverty, housing, and early child development and care. In further support of BI, our results show that immediate improvements in housing stability were accompanied by improvements in mental health and reduced stress, aligning with findings from previous studies that suggest income support can mitigate the psychological toll of poverty [20,29].

This study draws attention to the need to support participants in at the end of BI programs—a theme consistent across GI studies that report that families often struggle to sustain initial financial stability [14,17,30]. While financial planning workshops were offered by the county, few participants attended and most were unsuccessful in budgeting and saving in anticipation of the end of the program. Many participants struggled to wean their households off BI, and they sought more financial planning in retrospect. This study draws attention to the stressful mental load of BI programs ending and highlights how participants must creatively reassess their reentry into poverty and housing instability. Our qualitative methods draw out participant-driven suggestions for additional research to explore whether and how a longer duration of GI projects and/or additional supplementary support services could better enable families to ideate and maintain stability after the intervention ends.

We join previous BI research in calling for future programs to create and evaluate transition strategies, including extended housing assistance, ongoing case management, and access to affordable housing resources, which can help prevent participants from slipping back into instability [17,31]. While many BI programs incorporate financial literacy components [32,33], participants highlight the challenge of making them relevant and desirable so they can manage their stipends more effectively and plan for long-term stability. While existing evidence on GI and BI has not been able to demonstrate a causal relationship between financial education and improved economic outcomes for participants when programs end, the narratives in this research underscore that limited-term BI alone may not be enough to sustain benefits without additional programming to help participants transition.

Off-ramp planning is especially important for extending the realized benefits of BI for children. Before the program, participants reported that economic hardship directly and negatively impacted their desired parenting practices and the wellbeing of their children. Participant descriptions aligned with the FMS model [10], which highlights how daily financial stressors mediate between poverty and family processes such that a BI intervention theoretically opens an opportunity to shift these dynamics. Interviews revealed that many participants grew up in multi-generational poverty households. Having always lived in poverty, they took advantage of the opportunity to escape poverty, reporting that BI offered them stability and support, often for the first time in their lives. Mirroring other BI programs, families reported being able to cover essential costs such as rent, utilities, and other basic needs and move out of homelessness or insecure housing [14]. BI also helped to reduce financial uncertainty, leading to improvements in emotional and physical wellbeing, similar to the benefits identified in other BI pilot studies [31].

Unlike many quantitative or econometric-focused studies, our qualitative research draws attention to the greatest benefits reported by participants: more time with their children. While such time is difficult to quantify or qualify, especially in the critical window of early childhood development, participants foregrounded that additional time with their children was critical. The parents in the program who had children with special needs, including ADHD, learning differences, autism, genetic disorders, or other medical needs, found BI and the time it afforded them particularly helpful. Though YoBI aimed primarily to improve early childhood health and wellness, one important byproduct was improving the health and wellbeing of the parent. Our findings highlight that overall, BI funds changed their lives for the better, with many people emphasizing the emotional benefits which are difficult to quantify, underscoring the need for more qualitative methods in evaluating poverty alleviation programming and pathways.

**Figure 1:**
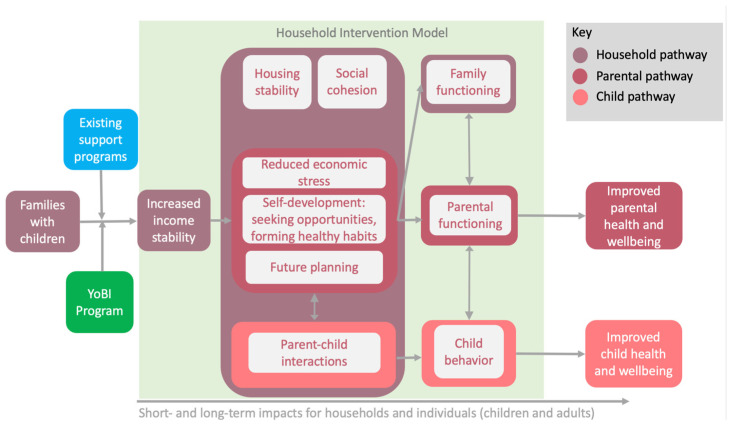
Emergent Theory of Change.

Based on participant input and the quantitative results presented elsewhere in the literature [34,35,36], the research team derived a Theory of Change for how BI interacts with the FSM and existing social services programming (Figure 1). This model helps bring FSM theory into conversation with traditional social services programming and BI pilot programming. Practically, participants expressed interest in combining BI and social services programming, emphasizing that the cash benefit was augmented by additional support and services. While participants universally expressed appreciation for unrestricted financial resources and were not asking for advice on how to spend their money, they readily acknowledged their lack of knowledge, skills and experience in the daunting task of managing and saving funds while living near the poverty line. To this end, we hope the YoBI Theory of Change is useful for future programming that seeks to further improve and quantify the impacts of BI on early childhood development.

To build on the successes of the YoBI program and ensure that families can maintain stability in the long run, two recommendations emerge from participant interviews. First, participants reported benefitting from case management and supplemental services such as job training, childcare support, and mental health services. These services helped participants build skills and resilience needed to sustain improvements in their financial situation and overall wellbeing. Previous studies similarly emphasize the importance of comprehensive support systems to accompany BI initiatives, ensuring that families receive the holistic support necessary for long-term success [30,36].

Second, while the short-term stability provided by BI was clear, it remains uncertain whether these early benefits will translate into long-term improvements in children’s health, educational outcomes, and overall wellbeing in years to come. Previous research has highlighted the importance of early interventions for improving life outcomes [23], emphasizing the critical development window of the first five years of life [37,38,39], but longitudinal studies are needed to assess the sustained effects of BI—if any. We recommend that future studies track BI-supported children over the long term to evaluate any lasting effects of early childhood financial stability on health, education, and overall wellbeing [20]. Such findings will help build a case for early childhood interventions while comparing BI approaches to more traditional child poverty alleviation programming.

## 5. Conclusions

This study highlights the multifactorial aspects of living in poverty, diverse uses of BI to mitigate poverty, and concerns with ending the BI program and re-entering poverty. Participants indicate that BI offers a valuable reprieve from poverty and reported using the funds to stabilize their housing and support the health and development of themselves and their children. This study demonstrated how BI coupled with robust case management temporarily reverses the dynamic described by the FSM, as cash assistance temporarily reduces stress, reduces caregiver mental load, and benefits early childhood development. While these benefits were clear when people received the payments, we recommend that future studies track BI-supported children and families over the long term to evaluate any lasting effects on housing, health, and overall wellbeing.

## Figures and Tables

**Table 1:** Self-reported demographic summary of 76 YoBI participants.

Age	21–55 Years Old (Average 32.8 Years)
Gender	
Female	92%
Male	8%
Primary language	
English	83%
Spanish	9%
Other (Russian, Farsi, Hmong)	8%
Ethnicity	
Hispanic/Latino	30%
Non-Hispanic/Latino	51%
Unknown	18%
Race	
Black or AA	24%
White	30%
Unknown	20%
Asian (including Hmong)	5%
Mexican, Puerto Rican, other Latin	20%
Other	1%
Yolo County Basic Income only	40%
Yolo County Basic Income + Housing Support Program	61%

## Data Availability

The data presented in this study are available on request from the corresponding author due to restrictions related to privacy.

## Abbreviations

The following abbreviations are used in this manuscript:

YoBI	Yolo County Basic Income
GI	Guaranteed Income
BI	Basic Income
CalWORKS	California Work Opportunity and Responsibility to Kids
HSP	Housing Support Services
